# Zfp423 Regulates Skeletal Muscle Regeneration and Proliferation

**DOI:** 10.1128/MCB.00447-18

**Published:** 2019-04-02

**Authors:** William N. Addison, Katherine C. Hall, Shoichiro Kokabu, Takuma Matsubara, Martin M. Fu, Francesca Gori, Roland Baron

**Affiliations:** a Department of Medicine, Harvard Medical School, Boston, Massachusetts, USA; b Department of Oral Medicine, Infection, and Immunity, Harvard School of Dental Medicine, Boston, Massachusetts, USA; c Division of Molecular Signaling and Biochemistry, Department of Health Promotion, Kyushu Dental University, Kitakyushu, Fukuoka, Japan; d Endocrine Unit, Massachusetts General Hospital, Harvard Medical School, Boston, Massachusetts, USA

**Keywords:** cell fate, myoblast, myogenesis, regeneration, satellite cells, skeletal muscle

## Abstract

Satellite cells (SCs) are skeletal muscle stem cells that proliferate in response to injury and provide myogenic precursors for growth and repair. Zfp423 is a transcriptional cofactor expressed in multiple immature cell populations, such as neuronal precursors, mesenchymal stem cells, and preadipocytes, where it regulates lineage allocation, proliferation, and differentiation. Here, we show that Zfp423 regulates myogenic progression during muscle regeneration. Zfp423 is undetectable in quiescent SCs but becomes expressed during SC activation. After expansion, Zfp423 is gradually downregulated as committed SCs terminally differentiate. Mice with satellite-cell-specific Zfp423 deletion exhibit severely impaired muscle regeneration following injury, with aberrant SC expansion, defective cell cycle exit, and failure to transition efficiently from the proliferative stage toward commitment. Consistent with a cell-autonomous role of Zfp423, shRNA-mediated knockdown of Zfp423 in myoblasts inhibits differentiation. Surprisingly, forced expression of Zfp423 in myoblasts induces differentiation into adipocytes and arrests myogenesis. Affinity purification of Zfp423 in myoblasts identified Satb2 as a nuclear partner of Zfp423 that cooperatively enhances Zfp423 transcriptional activity, which in turn affects myoblast differentiation. In conclusion, by controlling SC expansion and proliferation, Zfp423 is essential for muscle regeneration. Tight regulation of Zfp423 expression is essential for normal progression of muscle progenitors from proliferation to differentiation.

## Abstract (precis)

Satellite cells (SCs) are skeletal muscle stem cells that proliferate in response to injury and provide myogenic precursors for growth and repair. Zfp423 is a transcriptional cofactor expressed in multiple immature cell populations, such as neuronal precursors, mesenchymal stem cells, and preadipocytes, where it regulates lineage allocation, proliferation, and differentiation.

## INTRODUCTION

Osteoblasts, adipocytes, and myoblasts are derived from a common multipotent precursor known as the mesenchymal stem cell (MSC) (1). In addition to being able to differentiate into bone-, fat-, or muscle-forming cells, MSCs also have the capacity to self-renew. Multiple studies have demonstrated that cell fate decisions are mutually exclusive such that commitment to one lineage occurs at the expense of another. Imbalances or disruptions in the specification of alternate fates may contribute to several human diseases. For example, decreased osteogenesis accompanied by increased adipogenesis occurs during age-related osteoporosis and, conversely, ectopic bone formation in place of adipose tissue or muscle is observed in progressive osseous hyperplasia and fibrodysplasia ossificans progressiva (2–4). Recent lineage-tracing studies have also revealed a bidirectional cell fate switch between skeletal myoblasts and brown fat cells (5, 6). This is often the result of reciprocal transcriptional regulation of osteogenic, adipogenic and myogenic factors. For instance, the adipogenic transcription factor Pparγ inhibits the osteogenic factor Runx2 (7), and the pro-osteogenic factor Taz, while promoting Runx2 activity, inhibits Pparγ activity (8). Other proteins, such as ΔFosB and β-catenin, also have reciprocal effects on adipogenesis and osteogenesis (9–11). Furthermore, knockdown of the brown adipocyte transcription factor Prdm16 promotes the myogenesis of adipocytes, whereas overexpression of Prdm16 in myoblasts induces differentiation into brown adipocytes (5).

Although master regulators of differentiation have been well studied, the mechanisms governing early cell fate decisions are not entirely elucidated. Recently, we have shown that a novel family of transcription factors, Zfp423 and Zfp521, act as key regulators of the osteoblast versus adipocyte cell fate choice in MSCs (12, 13). Both are nucleus-localized transcriptional regulators containing 30 C2H2 type zinc fingers that play an important role in many biologic processes, including neurogenesis (14–16), hematopoiesis (17–19), osteoblastogenesis (12, 20–22), and adipogenesis (23, 24). Zfp521 induce MSCs to differentiate into osteoblasts, in part by repressing the expression of Zfp423, which is essential to promote adipogenesis (12, 13, 22, 23, 25). Zfp423 acts upstream of the adipogenic master regulator Pparγ to initiate a transcriptional cascade directing adipocyte lineage commitment (23). Forced expression of Zfp423 in nonadipogenic fibroblasts induces adipogenesis, and *in vivo* deletion of Zfp423 blocks fat formation (23). Whether or not Zfp423 also regulates the myoblast versus adipocyte “switch” remains unknown.

The cell fate decision of adult stem cells is particularly critical for skeletal muscle, due to its considerable potential for repair and regeneration following injury or disease (26–28). Muscle regeneration is a multistaged process mediated by a population of adult stem cells, positioned beneath the myofiber’s basal lamina, called satellite cells (26–28). Satellite cells are mitotically quiescent in healthy adult muscle, but upon muscle injury activated satellite cells reenter the cell cycle and proliferate extensively to form a pool of myoblasts, which then differentiate and fuse into new multinucleated myotubes (26–28). A subpopulation of satellite cell progeny resulting from asymmetric cell divisions also returns to a quiescent state to replenish the stem cell pool (26–28). Satellite cell functions involve a precise choreography of extracellular signaling cues and transcription factors that regulate gene expression networks to maintain quiescence, govern cell cycle reentry, or initiate a myogenic differentiation program. Quiescent satellite cells express paired box 7 (Pax7), whereas activated satellite cells and differentiating myogenic precursors also express the master transcription factor MyoD and other myogenic regulatory factors, such as the basic helix-loop-helix transcription factors Myf5 and myogenin (29–31). These myogenic regulatory factors bind regulatory elements of muscle-related structural genes, cell cycle-related genes, and other myogenic transcription factors to control differentiation during embryogenic myogenesis and adult muscle regeneration. Although numerous recent studies have improved our understanding of the signaling networks important for satellite function, the underlying mechanisms determining how satellite cell fate and transitions, self-renewal, and differentiation are regulated are poorly understood. These key questions are, however, central to future therapeutic interventions in muscle pathologies and regenerative medicine. Zfp423 expression is particularly abundant in immature cell populations such as neuronal and glial precursors in the developing brain, olfactory precursors, B-cell progenitors, and preadipocytes (14, 15, 23, 32, 33). In all of these cell types, Zfp423 functions as a regulator of lineage progression, differentiation, or proliferation. Zfp423 exerts these functions, at least in part, by physically interacting with other transcriptional coregulators such as Zfp521 (13) Ebfs (16, 34, 35), Smads (12, 23, 35), and Notch (36) to coordinate transcriptional activity downstream of several signaling pathways, including the bone morphogenetic protein (BMP), Notch, and Sonic hedgehog (Shh) pathways (37). In Zfp423-null mice, adipose tissue (23, 24) and cerebellum development (14, 15) are dramatically impaired. In humans, mutations of ZNF423 are linked to defects in DNA damage response and primary cilium function which together results in renal-related ciliopathies or Joubert’s syndrome (38, 39). Given that Zfp423 is involved in lineage progression in multiple tissues, and taking these results together with our studies showing that in mesenchymal stem cells Zfp423/Zfp521 interactions alter cell fate decisions, we hypothesized that Zfp423 could be a factor regulating early events in muscle stem cell function.

In the present study, we describe a novel role for Zfp423 as a regulator of skeletal muscle differentiation and regeneration. Zfp423 is expressed upon activation of satellite cells and is transcriptionally suppressed during the progression of myogenesis. Conditional deletion of Zfp423 in satellite cells using the *Pax7-Cre* driver, impairs muscle regeneration, and Zfp423 plays a critical role in the transition between satellite cell proliferation and myogenic differentiation.

## RESULTS

### Zfp423 is expressed in activated satellite cells.

Single myofiber isolation and culture preserve satellite cells in their physiological niche beneath the basal lamina and adjacent myofibers. To determine whether Zfp423 protein is expressed in quiescent and/or activated satellite cells, freshly isolated myofibers or suspension-cultured myofibers were analyzed by immunofluorescence staining.

**FIG 1:**
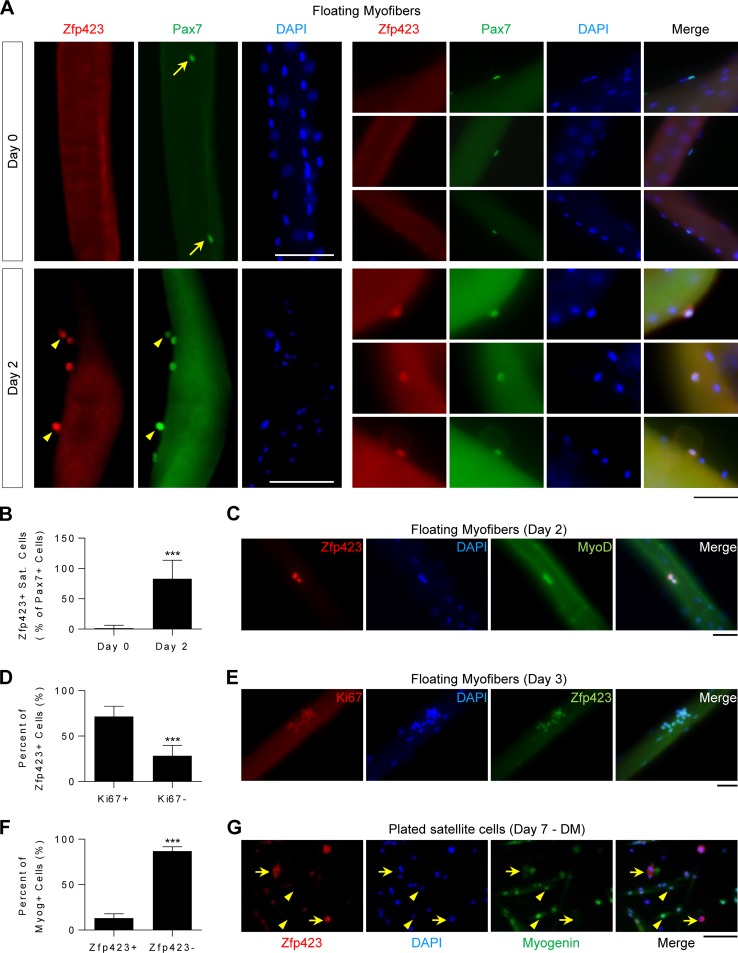
Zfp423 is expressed during satellite cell activation. (A to E) Freshly isolated myofibers, along with satellite cells in their niche, were immediately fixed (day 0) or suspension cultured in mitogen-rich activation medium for 2 to 3 days before fixation and immunostaining for Zfp423 and Pax7, MyoD, or Ki67. (A) Arrows indicate Zfp423-negative, Pax7-positive nuclei. Arrowheads indicate satellite cells doubly positive for both Zfp423 and Pax7. (B) Quantification of the number of Zfp423-positive satellite cells in the on days 0 and 2 of myofiber culture. (D) Quantification of the percentage of Zfp423-positive cells that express Ki67, as illustrated in panel E. (F and G) Satellite cells plated and differentiated for 7 days were stained for myogenin and Zfp423. The percentage of terminally differentiated (myogenin-positive) cells that express Zfp423 was quantified (F), and cells were imaged (G). (G) Arrows indicate Zfp423-positive, myogenin-positive cells, and arrowheads indicate Zfp423-positive, myogenin-negative cells. The data are presented as means ± the SD. ***, *P* < 0.001 (Student's *t* test). A total of >100 fibers were analyzed. Scale bars: 40 μm (A, C, and E) and 100 μm (G).

As shown in Fig. 1A, Zfp423 was undetectable in quiescent Pax7^+^ satellite cells on freshly isolated (day 0) myofibers. After stimulation with mitogen-rich medium (10% horse serum) for 48 h, activated Pax7^+^ satellite cells are marked by the expression of MyoD and the proliferation marker Ki67. As shown in Fig. 1A (lower panels), and in contrast to day 0, Zfp423 was readily detectable on activated Pax7^+^ satellite cells on day 2 of culture. Quantification of Zfp423^+^ satellite cell numbers confirmed that Zfp423 becomes expressed on activated Pax7^+^ satellite cells on day 2 (Fig. 1B). MyoD^+^ (Fig. 1C) and Ki67^+^ (Fig. 1E) cells also expressed Zfp423 at days 2 and 3, respectively. The majority of Zfp423^+^ cells were positive for Ki67 (Fig. 1D). However, Zfp423 was barely expressed in terminally differentiated myotubes since in Myog^+^ terminally differentiated satellite cells were rarely Zfp423^+^ (Fig. 1F and G). Taken together, these data indicate that Zfp423 is transiently expressed during myoblast differentiation: it is undetectable in quiescent satellite cells but becomes expressed in satellite cells after activation and is later repressed during progression toward a mature cell type.

### Zfp423 expression is downregulated during myogenesis.

**FIG 2:**
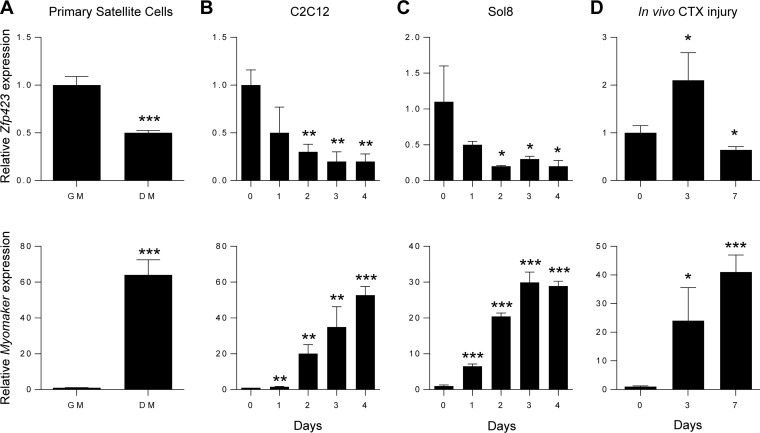
Zfp423 expression is downregulated during myogenic differentiation. (A) Primary satellite cells were plated and cultured in adherent culture conditions in growth medium (GM) or differentiation medium (DM) for 3 days, after which qRT-PCR analysis of *Zfp423* and *Myomaker* expression was performed. (B) qRT-PCR of *Zfp423* and *Myomaker* gene expression in undifferentiated proliferating C2C12 cells (day 0) and over the course of 4 days after the induction of myoblast differentiation. (C) qRT-PCR of *Zfp423* and *Myomaker* expression in Sol8 myoblast cells during differentiation for the indicated number of days. (D) qRT-PCR showing *Zfp423* and *Myomaker* expression during the course of muscle regeneration. TA muscles were injected with cardiotoxin (CTX) and harvested on the indicated days after injury for RNA. The data are presented as means ± the SD (*n* ≥ 3). *, *P* < 0.05; **, *P* < 0.01; ***, *P* < 0.001 (relative to GM or day 0; Student's *t* test).

The presence of Zfp423 in activated satellite cells combined with its absence in differentiated myotube nuclei suggests that Zfp423 is downregulated during myogenic differentiation. To determine whether *Zfp423* expression was differentially regulated during myogenesis, we examined mRNA levels of *Zfp423* in various cell culture models of myoblast differentiation, including primary satellite cells, Sol8, and C2C12 cells. Primary satellite cells plated in adherent culture conditions were induced to differentiate into myotubes by stimulating cells with differentiation medium for 3 days. qPCR analysis showed that *Zfp423* was expressed in proliferating undifferentiated satellite cells and downregulated after myotube formation (Fig. 2A), as indicated by an inverse correlation with the expression of the myoblast fusion gene, *Myomaker*. We next examined *Zfp423* levels in two well-established myoblast cell lines. As shown in Fig. 2B and C, *Zfp423* mRNA in Sol8 or C2C12 cells was highest during the proliferating or undifferentiated stage of myoblast precursors and steadily declined during myogenic differentiation.

Taken together, these data suggest that *Zfp423* expression levels are linked to the progression of myogenic differentiation and that *Zfp423* may play a role in the proliferation and/or differentiation of myoblast cells.

### Zfp423 deletion impairs muscle regeneration *in vivo*.

Skeletal muscle regeneration mirrors much of the myoblast differentiation process. To determine whether Zfp423 is regulated during muscle regeneration *in vivo*, we monitored *Zfp423* expression during muscle healing after cardiotoxin (CTX)-induced muscle injury. As shown in Fig. 2D, *Zfp423* expression was increased by day 3 after muscle injury and then declined by day 7. The initial increase of *Zfp423* at day 3 coincides with the loss of muscle and the recruitment and expansion of satellite cells during the first 3 days of the healing process, whereas the reduction in expression between days 3 and 7 likely reflects the differentiation and fusion of myoblasts.

**FIG 3:**
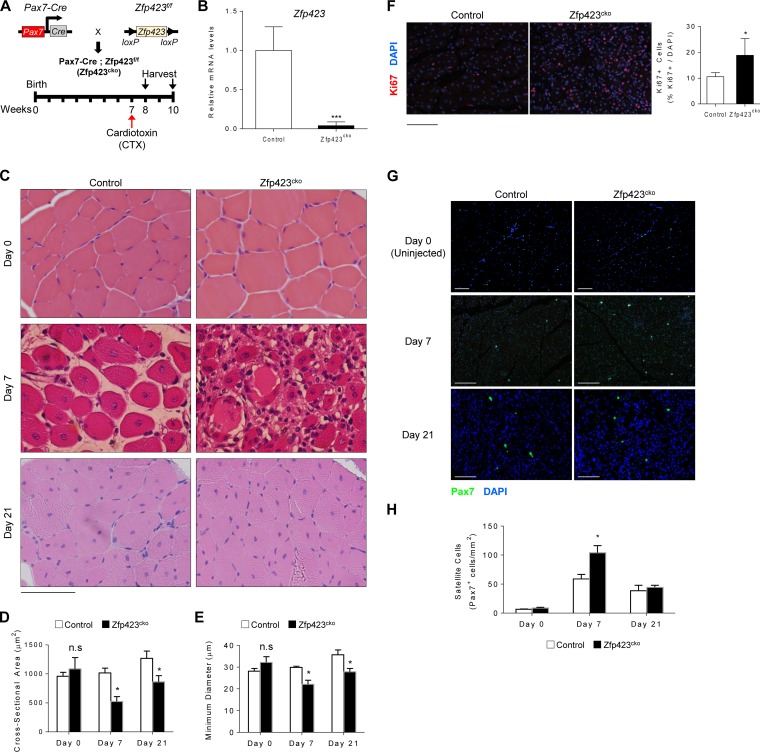
Zfp423 is critical for muscle regeneration *in vivo*. (A) Schematic of CTX injury model. (B) Expression of *Zfp423* in TA muscles of 7-week-old *Pax7^Cre^*; *Zfp423^f/f^* (Zfp423^cko^) mice. (C) H&E staining of TA muscles from control and Zfp423^cko^ mice in uninjured conditions (day 0), as well as 7 and 21 days after injury. (D and E) Cross-sectional area (D) and minimal Feret’s diameter (E) of regenerating myofibers at the indicated time points following injury. (F) Ki67 immunostaining and quantification on control and Zfp423^cko^ TA muscles at day 7 after injury. (G and H) Pax7 immunostaining (G) and quantification on control and Zfp423^cko^ TA muscles (H) at days 0, 7, and 21 after injury. The data are presented as means ± the SD (*n* ≥ 3). n.s, not significant; *, *P* < 0.05; ***, *P* < 0.001 (Student's *t* test). Scale bars: 65 μm (C) and 100 μm (F and G).

Based on the differential expression pattern of *Zfp423* during myogenesis and muscle regeneration, we sought to determine whether Zfp423 might play a role in muscle regeneration *in vivo*. To this aim, we generated mice with a satellite cell-specific deletion of Zfp423 by crossing *Zfp423^f/f^* mice with *Pax7^Cre^* and injected CTX into the tibialis anterior (TA) muscle to induce skeletal muscle injury. Muscle regeneration was assessed following the scheme illustrated in Fig. 3A. qPCR analysis of TA muscle tissue from control (*Zfp423^f/f^*) and *Pax7^Cre^*; *Zfp423^f/f^* conditional knockout (Zfp423^cko^), mice showed that Zfp423 expression was efficiently depleted (>96%) after Cre excision (Fig. 3B). Zfp323^cko^ mice showed no overt morphological abnormality in the absence of injury. TA muscles of control or Zfp423^cko^ mice were injected at 7 weeks of age and muscles isolated for histological evaluations 7 and 21 days after injection. As expected, 7 days after injury, control mice displayed an efficient regenerative response typified by numerous large centrally nucleated regenerated myofibers (Fig. 3C). In contrast, injured Zfp423^cko^ muscles contained an abundance of fibrotic tissue, inflammatory cells, and damaged myofibers with multiple peripheral nuclei (Fig. 3C). At 21 days after injury, muscle damage and fibrosis were largely cleared in both control and Zfp423^cko^ mice (Fig. 3C). However, Zfp423^cko^ muscle fibers remained were slightly smaller in cross-sectional area and minimum diameter (Fig. 3D and E). Together, these observations suggest that Zfp423 is required for proper muscle regeneration.

Muscle regeneration requires an initial proliferation and expansion of satellite cells prior to differentiation. To assess whether proliferation and expansion are affected in Zfp423^cko^ mice, we quantified the number of Ki67^+^ nuclei (a marker of proliferative cells) following CTX injury. As seen in Fig. 3F, the number of proliferative cells was significantly increased in Zfp423^cko^ mice compared to control mice after injury. Furthermore, this accumulation of proliferative cells was accompanied by a significant and transient increase in the number of Pax7^+^ satellite cells in injured Zfp423^cko^ muscle compared to control mice (Fig. 3G and H). Taken together with the observed regeneration defect, the data strongly suggest that Zfp423-deficient satellite cells fail to transition efficiently out of the proliferative phase into a differentiation stage during myofiber regeneration.

### Zfp423 controls satellite cell proliferation.

**FIG 4:**
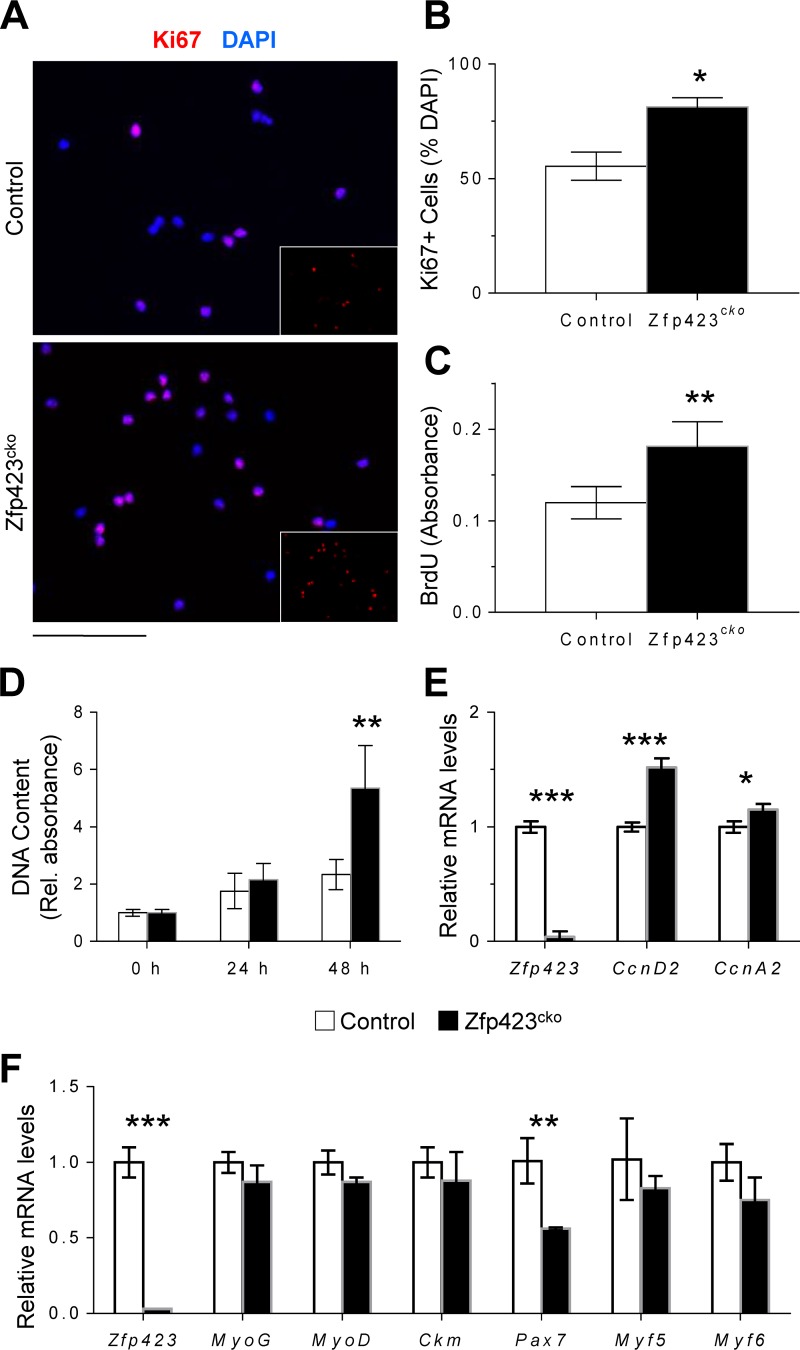
Zfp423 impairs proliferative ability in satellite cells. (A to C) MACS-isolated wild-type and Zfp423^cko^ satellite cells were immunostained and quantified for Ki67 and DAPI (A and B) or labeled with BrdU for 5 h for quantification of BrdU incorporation by anti-BrdU ELISA (C). (D) Wild-type and Zfp423^cko^ satellite cells were plated and grown for 72 h, and proliferation was assessed by DNA quantification. (E) qRT-PCR of cell cycle regulators *cyclin D2* and *cyclin A2* in satellite cells grown as for panel D. (F) qRT-PCR of wild-type and Zfp423^cko^ satellite cells cultured in differentiation medium (DM) for 3 days. The data are presented as means ± the SD (*n* ≥ 3). *, *P* < 0.05; **, *P* < 0.01; ***, *P* < 0.001 (Student's *t* test). Scale bar, 40 μm.

In principle, Zfp423 could be involved in the activation, proliferation and/or differentiation of satellite cells during injury. To identify the Zfp423-dependent step and to investigate the cellular mechanisms underlying the impaired muscle regeneration and accumulation of proliferative cells in Zfp423^cko^ mice, we isolated and analyzed magnetic cell sorting (MACS)-purified satellite cells from Zfp423^cko^ and control mice. Zfp423-deficient satellite cells showed an increase in the percentage of proliferating cells, as reflected by quantification of Ki67^+^ nuclei (Fig. 4A and B). Consistent with this observation, Zfp423^cko^ cells synthesized DNA at a higher rate, as indicated by increased bromodeoxyuridine (BrdU) incorporation (Fig. 4C). When cells were plated at equal densities, higher numbers of Zfp423^cko^ cells were observed after 48 h of culture (Fig. 4D). D-type cyclins are important elements of the cell cycle machinery and play a role in cell cycle progression and withdrawal in satellite cells (40). Consistent with the increased proliferation observations, expression of cyclin D2 was upregulated in Zfp423^cko^ cells (Fig. 4E). The levels of other D-type cyclins remained unchanged, suggesting a unique or distinct dysregulation of *Cyclin D2* in the absence of Zfp423.

We next compared the abilities of control and Zfp423^cko^ cells to differentiate in culture. As shown in Fig. 4F, after 3 days in differentiation medium, the expression of terminal differentiation markers such as the creatine kinase gene (*Ckm*) were unchanged between control and *Zfp423^ck^*^o^ cells. Moreover, deletion of Zfp423 did not significantly alter markers of specification such as *Myf5* or *MyoD* (Fig. 4F). *Pax7* expression was significantly downregulated, perhaps reflecting a faster exit from the stemness state (Fig. 4F). Thus, loss of Zfp423 *ex vivo* significantly alters myoblast proliferation without impairing myoblast differentiation.

### Forced expression of Zfp423 alters myoblast cell fate.

**FIG 5:**
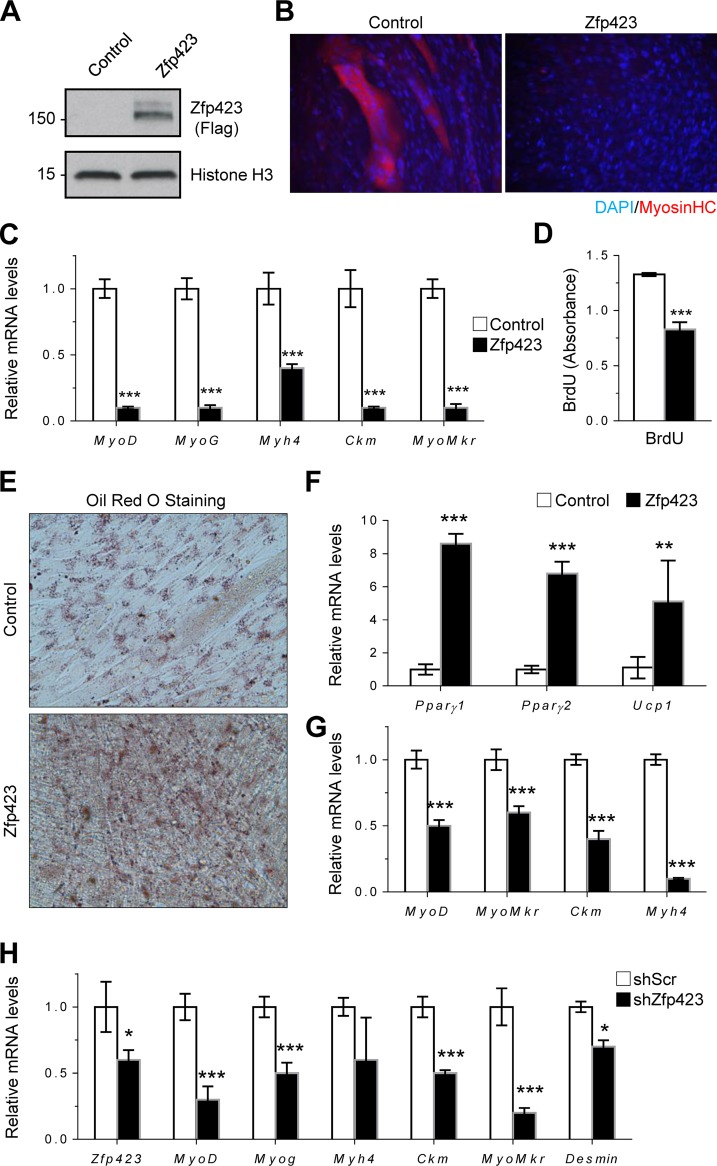
Forced expression of Zfp423 stimulates adipogenic differentiation in skeletal myoblasts. (A) Confirmation of Zfp423 overexpression by anti-FLAG Western blotting. (B) Sol8 cells stably expressing Zfp423 were induced to differentiate for 3 days, after which terminally differentiated myotubes were visualized by anti-MHC immunostaining. (C) qRT-PCR of myogenesis-selective genes in the differentiated cultures shown in panel B. (D) Sol8 cells stably expressing Zfp423 were labeled with BrdU for 5 h, and BrdU incorporation was quantified by anti-BrdU ELISA. (E and F) Sol8 cells stably expressing Zfp423 were cultured in adipogenic differentiation medium for 6 days, after which Oil Red O staining of lipid accumulation (E) or qRT-PCR analysis of adipocyte-specific genes (F) was performed. (G) qRT-PCR of myoblast-specific genes in the cultures described in panels D and E. (H) Sol8 cells expressing Zfp423 shRNA were cultured in myogenic differentiation medium for 3 days, after which myogenesis-selective genes were measured by qRT-PCR. The data are presented as means ± the SD (*n* ≥ 3). *, *P* < 0.05; **, *P* < 0.01; ***, *P* < 0.001 (Student's *t* test).

As observed previously, *Zfp423* expression declines during myoblast differentiation, suggesting that the regulated reduction of Zfp423 may be required for the normal progression of myoblast differentiation. To determine whether this is the case, we stably overexpressed Zfp423 in Sol8 myoblasts and examined their myogenic potential *in vitro*. Sol8 cells stably transfected with FLAG-tagged Zfp423 expression vector expressed a high level of Zfp423 protein (Fig. 5A). In response to myogenic differentiation medium, Sol8 cells expressing control vector differentiated proficiently into multinucleated myosin heavy chain (MHC)-expressing myotubes, whereas Zfp423-expressing Sol8 cells failed to undergo significant terminal myocyte differentiation (Fig. 5B). In addition, gene expression analysis by qPCR indicated that Zfp423 expression led to a significant reduction in the expression of myogenic-specific genes (Fig. 5C). Furthermore, cell proliferation was decreased following Zfp423 overexpression, as assessed by BrdU incorporation (Fig. 5D), consistent with our previous observation of increased proliferation in Zfp423^cko^ cells (Fig. 4C).

Since myoblasts and brown adipocytes have been shown to be derived from a common precursor during development and since Zfp423 is important for adipogenic differentiation, we hypothesized that the inhibition of myogenesis by Zfp423 might be related to the specification of myoblast cells toward an adipogenic fate by Zfp423. To investigate whether Zfp423 confers adipogenic differentiation potential to myoblast cells, control and Zfp423-expressing Sol8 cells were cultured in adipogenic differentiation medium. In response to the proadipogenic culture conditions, Zfp423-expressing cells underwent robust adipogenic differentiation, as indicated by a massive accumulation of Oil Red O-stained lipid droplets (Fig. 5E), as well as an upregulation of adipocyte-specific genes such as *Pparγ* and the brown adipocyte-specific gene *Ucp1* (Fig. 5F). Upregulation of adipocyte-specific genes in Zfp423-expressing cells was concomitant with a significant downregulation of myoblast-lineage markers (Fig. 5G), which is indicative of a bona fide adipogenic conversion of the Sol8 myoblast cell line. We next examined the requirement for Zfp423 in Sol8 myoblast differentiation. shRNA-mediated knockdown of *Zfp423* expression significantly impaired myogenesis, as indicated by a lower expression of myoblast-selective genes (Fig. 5H).

**FIG 6:**
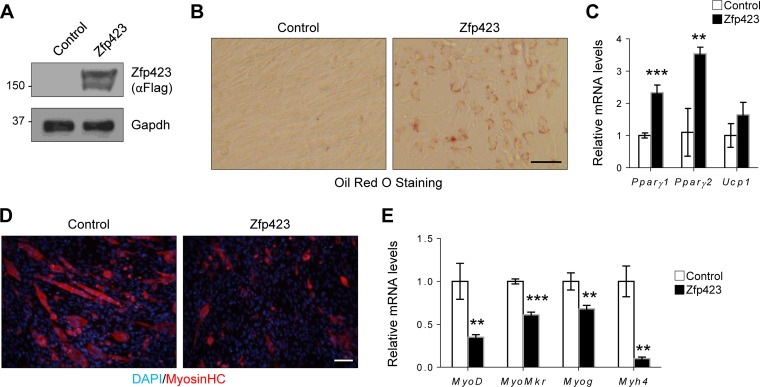
Zfp423 stimulates adipogenic differentiation in C2C12 myoblasts. (A) Confirmation of Zfp423 overexpression by anti-FLAG Western blotting. (B and C) C2C12 cells stably expressing Zfp423 were cultured in adipogenic differentiation medium for 6 days, after which Oil Red O staining of lipid accumulation (B) or qRT-PCR analysis of adipocyte-specific genes (C) was performed. (D and E) C2C12 cells stably expressing Zfp423 were cultured in myogenic differentiation medium for 3 days, after which terminally differentiated myotubes were visualized by anti-MHC immunostaining (D) and myogenesis-selective genes were analyzed by qRT-PCR (E). The data are presented as means ± the SD (*n* ≥ 3). **, *P* < 0.01; ***, *P* < 0.001 (Student's *t* test). Scale bars, 100 μm.

Consistent with our observation in Sol8 cells, ectopic expression of Zfp423 in C2C12 cells was also sufficient to promote adipogenesis (Fig. 6). Oil Red O staining (Fig. 6B) and gene expression analysis (Fig. 6C) showed that Zfp423-expressing myoblast cells will undergo robust adipogenic differentiation under proadipogenic conditions. Furthermore, Zfp423-expressing C2C12 cells failed to undergo robust myogenesis (Fig. 6D and E). We conclude that sustained or ectopic Zfp423 expression activates an adipogenic transcriptional program in myoblasts, and thus *Zfp423* must be downregulated during myogenic differentiation.

Therefore, both forced Zfp423 expression and *Zfp423* knockdown in the committed myoblast cell line resulted in impaired differentiation. Thus, unlike in satellite cells where Zfp423 was mostly dispensable for differentiation, a tight regulation of Zfp423 levels is critical for the myogenesis of committed myoblasts.

### Zfp423 binds Satb2 to cooperatively regulate transcription in C2C12 cells.

**FIG 7:**
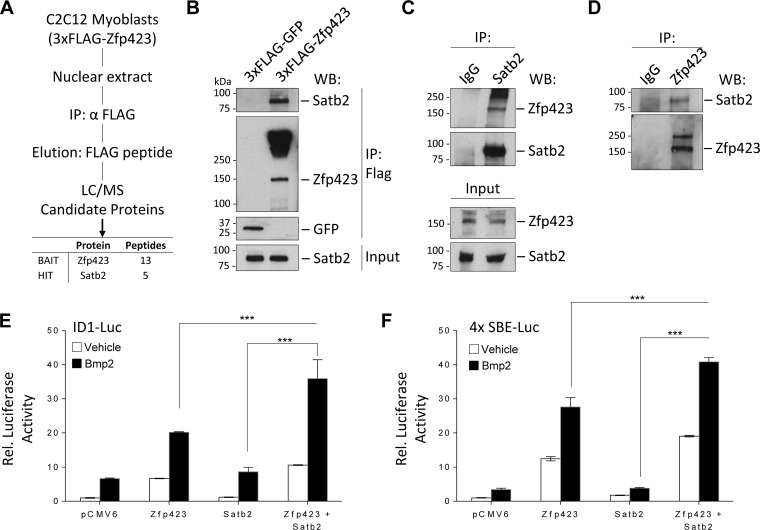
Zfp423 associates with Satb2 to cooperatively regulate transcription. (A) Schematic overview of the proteomics approach used to identify Zfp423 interacting proteins in C2C12 myoblast cells. FLAG-Zfp423 was purified from nuclear extracts with anti-FLAG antibody resin and analyzed by MS. See Table S1 in the supplemental material for a complete list of interactants. (B) Immunoprecipitation analysis of FLAG-Zfp423 with endogenous Satb2. C2C12 cells stably expressing FLAG-Zfp423 or FLAG-GFP were lysed and immunoprecipitated (IP) with anti-FLAG antibody and blotted (WB) with anti-FLAG antibody or anti-Satb2 antibody. (C and D) Confirmation of endogenous Zfp423 interaction with endogenous Satb2. Zfp423 or Satb2 was immunoprecipitated from untransfected C2C12 cells with anti-Zfp423 or anti-Satb2 antibody and immunoblotted with the indicated antibody. Naive IgG was used as a negative-control antibody. (E) Zfp423 works in concert with Satb2. The transcriptional activity of a Bmp-responsive Id1 promoter luciferase (ID1-Luc) construct in the presence of the indicated expression vectors for Zfp423 or Satb2 was assessed. (F) Luciferase activity of a BMP-responsive construct containing four tandem repeats of the SMAD binding elements (SBE-Luc) in cells transfected with the indicated expression vectors. All luciferase activity was measured 16 h after treatment with 100 ng/ml BMP2. relative to the empty-vector control unless otherwise indicated. The data are presented as means ± the SD (*n* ≥ 3). ***, *P* < 0.001 (Student's *t* test).

To explore possible mechanisms by which Zfp423 functions in muscle cells, we used an unbiased affinity purification and proteomics approach to identify binding partners of Zfp423. FLAG-tagged Zfp423 complexes were immunopurified from C2C12 myoblast cells and analyzed by mass spectrometry (MS) (Fig. 7A; see also Table S1 in the supplemental material). Of particular interest was the identification of special AT-rich sequence-binding protein 2 (Satb2), because of its known role as a BMP-induced gene and the direct Smad1/5 target gene (41). Satb2 is a DNA-binding nuclear matrix protein that participates in chromatin remodeling and transcriptional regulation (42). Satb2 is postulated to regulate cell-specific gene expression by tethering DNA elements, chromatin-remodeling complexes, and transcription factors (43). Independent immunoprecipitation assays of FLAG-Zfp423 in C2C12 cells showed endogenous Satb2 in a complex with FLAG-Zfp423 (Fig. 7B). We further validated the endogenous association of Zfp423 with endogenous Satb2 by performing immunoprecipitation with antibodies against Satb2 (Fig. 7C) and Zfp423 (Fig. 7D) in untransfected C2C12 cells. Similar observations were made in Sol8 cells (data not shown). Given that Zfp423 and Satb2 are transcriptional regulators, we next examined whether Zfp423 and Satb2 can coregulate transcription.

Interestingly, Zfp423 was the first identified Smad-interacting cofactor of the BMP signaling pathway (35). Previously, we showed that during BMP-induced differentiation of mesenchymal stem cells, the adipocyte lineage is suppressed by Zfp521 via a direct transcriptional repression of Zfp423 thus, providing a mechanism to fine tune MSC cell fate decision (12). BMP signaling is absolutely essential for satellite cell expansion and muscle regeneration (44–47). Deletion of Smad4 or its target Id1 in muscle cells impairs muscle regeneration (44–47). The association of the transcriptional regulator Satb2 with Zfp423 prompted us to investigate the role of Zfp423-Satb2 complexes in BMP-induced transcriptional activity. As shown in Fig. 7E, expression of Zfp423 stimulated the activity of a luciferase reporter controlled by the BMP responsive region from the Id1 promoter (IDWT4-luciferase [48]). Although Satb2 alone had no effect on luciferase activity, coexpression of Zfp423 and Satb2 further increased luciferase activity 35-fold, a finding indicative of a cooperative synergy between Zfp423 and Satb2. These observations were also confirmed with a luciferase construct containing four copies of the Smad binding elements (SBE4-luc [42]) (Fig. 7F). Thus, Zfp423 and Satb2 can interact both physically and transcriptionally.

### Satb2 regulates myoblast differentiation and proliferation.

**FIG 8:**
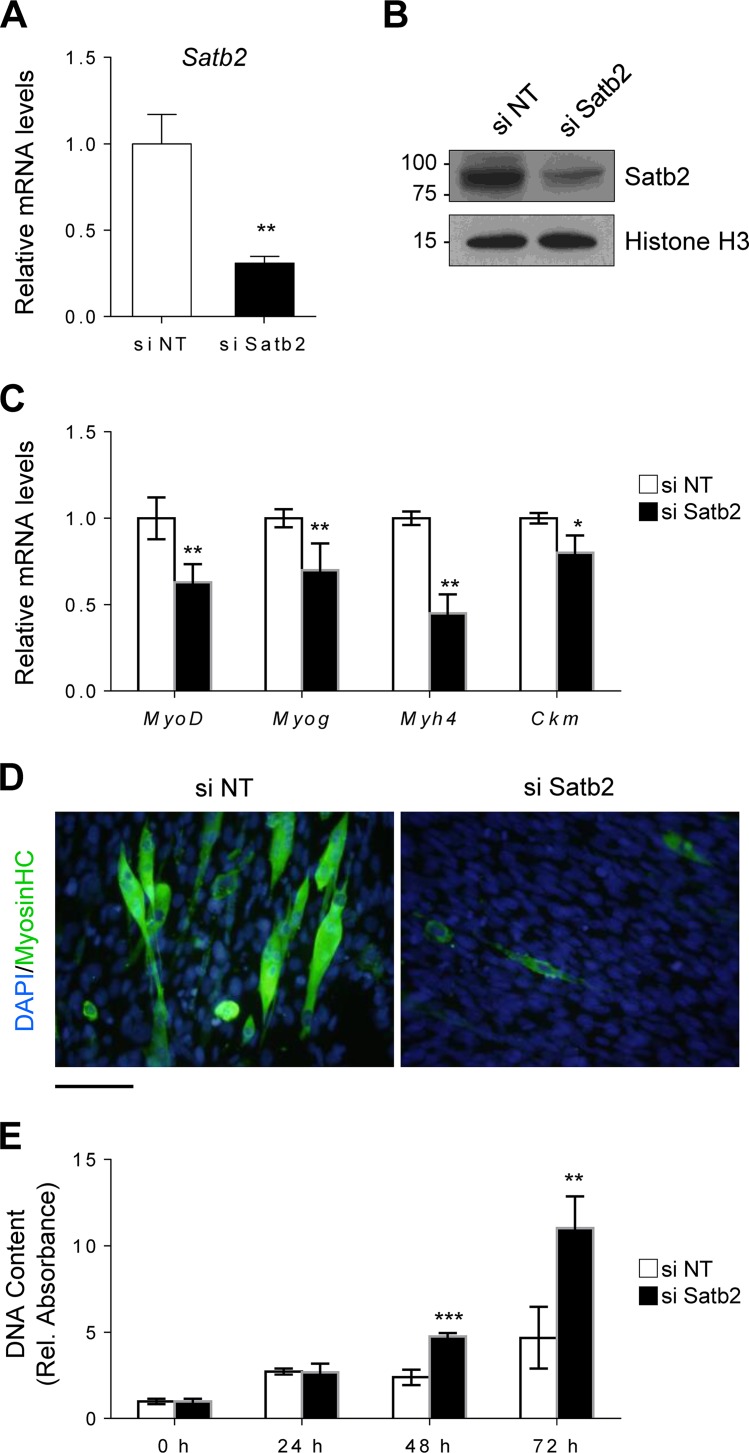
Satb2 regulates myoblast differentiation and proliferation. (A and B) Satb2 mRNA (A) and protein (B) were efficiently depleted by siRNA-mediated knockdown in Sol8 cells. Nontargeting siRNA (si NT) was used as a control. (C and D) Satb2 knockdown cells were induced to differentiate for 3 days, after which myogenesis-selective genes were analyzed by qRT-PCR (C) and terminally differentiated myotubes were visualized by anti-MHC immunostaining (D). (E) Satb2 knockdown cells and nontargeting siRNA control cells were seeded at a low density and cultured for 72 h, during which proliferation was monitored. The data are presented as means ± the SD (*n* ≥ 3). *, *P* < 0.05; **, *P* < 0.01; ***, *P* < 0.001 (Student's *t* test). Scale bar, 100 μm.

To understand the implications of a cooperative interaction between Zfp423 and Satb2, we next sought to determine the role of Satb2 in myogenic differentiation and proliferation. Using small interfering RNA (siRNA) targeted against Satb2, we knocked down Satb2 mRNA (Fig. 8A) and protein (Fig. 8B) expression in Sol8 cells. In response to myogenic differentiation medium, Satb2-depleted cells displayed reduced myogenic differentiation, as indicated by lower expression of myogenesis-selective genes (Fig. 8C) and MHC myotube staining (Fig. 8D). Furthermore, Satb2-knockdown cells exhibited an increased proliferative capacity compared to control cells (Fig. 8E). These data suggest that, similar to Zfp423, Satb2 is important for myogenic function and is required for normal differentiation and proliferation.

**FIG 9:**
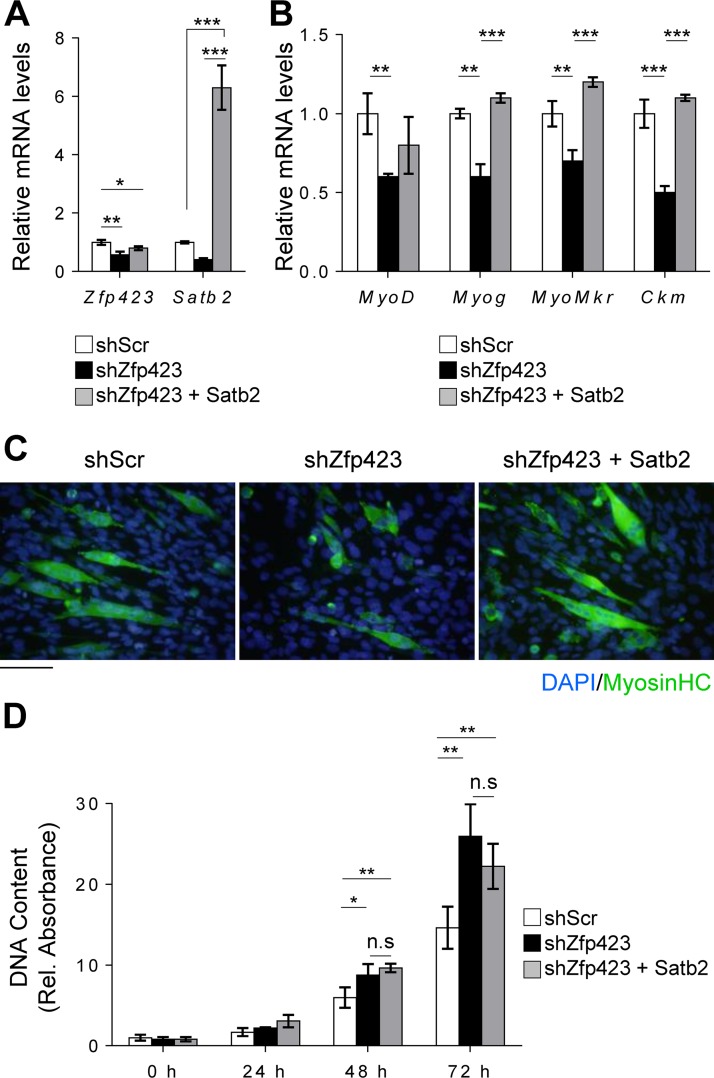
Functional interaction between Zfp423 and Satb2 in myogenesis. Sol8 cells stably expressing Zfp423 shRNA (shZfp423) or scrambled shRNA (shScr) were transfected with control (empty vector) or Satb2 expression vectors. (A) After selection, qRT-PCR confirmed the overexpression of *Satb2*. (B and C) Cells were then differentiated for 3 days in myogenic differentiation medium, and the expression of myogenesis-selective genes was quantified by qRT-PCR (B) and differentiated myotubes were visualized by anti-MHC immunostaining (C). (D) DNA content as a measure of cell proliferation. The data are presented as means ± the SD (*n* ≥ 3). *, *P* < 0.05; **, *P* < 0.01; ***, *P* < 0.001 (ANOVA with a Fisher least-significant-difference *post hoc* test). Scale bar, 100 μm.

To investigate whether Zfp423 function in myoblasts is mediated by Satb2, we overexpressed Satb2 in Zfp423-shRNA-expressing Sol 8 cells (Fig. 9A). Overexpression of Satb2 rescued and restored the differentiation potential of Zfp423-deficient Sol8 cells (Fig. 9B and C). The expression of the myogenic differentiation markers *Myog*, *MyoMkr*, and *Ckm* returned to the levels of control cells following Satb2 overexpression (Fig. 9B). However, Satb2 overexpression failed to rescue the increased proliferative ability of shZfp423 cells (Fig. 9D). This suggests that although Zfp423 function during differentiation is in part mediated by Satb2, its role in proliferation may not exclusively be via Satb2. Taken together, our data indicate that Zfp423 and Satb2 not only physically and functionally interact in skeletal muscle cells and cooperatively regulate certain aspects of myogenic differentiation but also act independently of each other in certain proliferative functions.

## DISCUSSION

**FIG 10:**
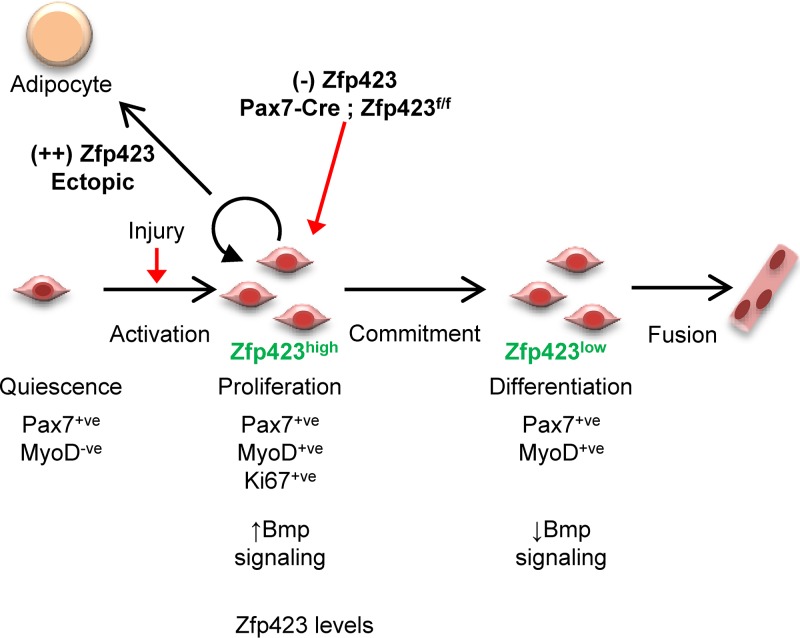
Model for the role of Zfp423 in adult muscle regeneration. During adult muscle regeneration, quiescent Pax7^+^ satellite cells do not express Zfp423, but activated cells begin to express Zfp423. After extensive proliferation, cells committed to myogenic differentiation downregulate Zfp423 and fuse to form myotubes. Loss of Zfp423 results in a delay of progression from the proliferative state to the myogenic differentiation state. Thus, Zfp423 controls the proliferation of adult satellite cells to affect muscle regeneration and myogenesis.

The *in vivo* and *in vitro* data presented in this study reveal a role for Zfp423 in the regulation of adult skeletal muscle differentiation and regeneration (Fig. 10). Following an acute injury, satellite cells are activated and Zfp423 expression is simultaneously induced. Activated satellite cells then proliferate to generate myoblasts which differentiate into new multinucleated myotubes as Zfp423 is concomitantly silenced. In the absence of Zfp423, activated satellite cells fail to progress efficiently from the proliferative phase into a differentiation state resulting in impaired muscle regeneration.

Our previous work on Zfp423 has demonstrated its importance in MSC cell fate decision between adipocytes and osteoblasts (12, 13, 20–22, 25, 49, 50). Strong repression of Zfp423 by Zfp521 in mesenchymal stem cells was critical for directing cell fate responses to BMP (12). In the bone microenvironment, Bmp2 induces osteoblast differentiation and bone formation (51, 52). When Zfp423 was derepressed by the absence of Zfp521, Bmp2 responses were directed toward adipocyte differentiation and fat formation (12). Here, we similarly demonstrate that in myoblast cells, forced expression of Zfp423 misdirects the progression of myoblast differentiation into a fat accumulating adipocyte-like phenotype. Consistent with our prior observations in mesenchymal stem cells, an intrinsic repression of Zfp423 is required in quiescent satellite cells prior to differentiation. It therefore appears that the expression of Zfp423 needs to be transiently allowed in order to direct precursor cells toward the myoblast phenotype. Our previous work suggests a model where continuous repression of Zfp423 is required for the osteoblast lineage and continuous expression for the adipocyte phenotype. Here, we show that a transient expression of Zfp423, followed by its repression, is required to acquire the myoblast phenotype.

To elucidate the molecular basis underlying Zfp423 function in muscle cells, we used a proteomics approach to identify Satb2 as a potential functional interactant for Zfp423. The Satb2 loss-of-function experiments presented here provide evidence that Satb2 also plays a role in myogenic differentiation and proliferation. These data are, to the best of our knowledge, the first such evidence for a role of Satb2 in myogenesis. Furthermore, since loss of Satb2 phenocopies the effect of loss of Zfp423, it is highly likely that Zfp423 and Satb2 function in concert to regulate certain aspects of myogenesis. Satb2 or Zfp423 knockdown results in decreased myogenic differentiation and increased proliferation. Indeed, overexpression of Satb2 in Zfp423-depleted cells was able to partially rescue the differentiation defect. Remarkably, Satb2 overexpression did not, however, prevent the increased proliferation observed with Zfp423 depletion. This decoupling of differentiation and proliferation suggests that although Satb2 is a major mechanism for Zfp423 function there are most likely other factors involved. A systematic analysis of the specific and shared targets of these two transcription factors will be an interesting approach to address this question.

The interaction of Zfp423 and Satb2 in myoblasts, along with the strong coactivation of BMP transcriptional activity, suggests a possible mechanism by which Zfp423 affects muscle genesis. By titrating levels of Zfp423 following satellite cell activation and expansion, BMP signaling intensity and duration can be subsequently controlled. Indeed, several lines of evidence indicate that the BMP/Smad signaling pathway is involved in a critical switch between proliferation and differentiation of satellite cells (45, 53) and the maintenance of adult muscle mass (44). BMP activity dynamics during muscle regeneration mirrors that of Zfp423 expression. BMP signaling is activated following muscle injury and satellite cell activation but is then reduced during myoblast differentiation (46, 47, 53, 54). BMP signaling maintains satellite cells in a proliferative state thereby enhancing expansion. In this study, we have described a dramatic temporal regulation of Zfp423 expression in satellite cells that might in turn allow for BMP signaling intensity and duration to be tightly controlled. Although our data do not directly indicate that Zp423-Satb2 interactions and BMP signaling are involved in our observed cellular phenotypes, our current understanding of the role of BMP signaling in myogenesis suggests that regulation of BMP signaling could explain our observations.

Since Zfp423 interacts with multiple transcription factors, it is nonetheless possible that Zfp423 function in muscle is also mediated by other factors. Zfp423’s mechanism of action in other tissues has also involved transcriptional regulation of Ebf (16, 34), Notch (36), and Shh signaling (37) through direct interaction with Ebf1 or the Notch1 intracellular domain (Nicd1). Although these pathways have been shown to be strongly linked to muscle regeneration and satellite cell function (55–58), our proteomics screen in myoblast cells did not detect Zfp423 interaction with Ebf1 or Nicd1. So, whereas Zfp423 interaction with Ebf1 or Nicd1 may be critical in neuronal cells, alternative pathways may be involved in muscle cells.

The role of Zfp423 in orchestrating cell proliferation has been described in B cells (18, 19), neuronal precursors (14, 15, 59, 60), and preadipocytes (23). In olfactory neuron precursors, sustained Zfp423 expression arrests cells in an immature undifferentiated state by sequestering Ebf1 (59, 61). In this way, synchronized transitions out of the cell cycle are tightly regulated by Zfp423 (14, 38). In the developing cerebellum, Zfp423 deficiency resulted in decreased proliferation within granule cell precursors and neuroglial cells (14, 15, 60). Altogether, these data suggested that in the absence of Zfp423, there is an abnormal coupling of differentiation and cell cycle exit in neural precursors similar to the dysregulation of proliferation and differentiation we have observed in skeletal muscle.

Given that Zfp423 is silenced during myoblast differentiation, we expected that forced expression would prevent myogenesis *in vitro*. Surprisingly, we also observed that increased Zfp423 expression converted myoblast into adipocytes *in vitro*. Several recent lineage tracing experiments have demonstrated that brown adipocytes and myoblast may share a precursor (5, 6, 62). Indeed, Prdm16, an Ebf and Zfp423 target, controls a brown fat/muscle lineage switch. Ucp1-expressing brown adipocytes have been observed in muscle tissue (63), and satellite cells can undergo adipogenesis under some experimental conditions (64, 65).

In conclusion, we have shown that Zfp423 regulates muscle regeneration *in vivo*, and we have identified Zfp423 as a novel regulator of satellite cell proliferation. We observed that Zfp423 is nearly undetectable is quiescent satellite cells. However, it is significantly upregulated following activation of satellite cells *in vitro* and *in vivo*. Furthermore, it is dramatically downregulated as differentiation proceeds. Our data suggest that Zfp423 regulates the transition between proliferation and differentiation. These data establish Zfp423 as an important player in satellite cell function and adds to the current knowledge of potentially exploitable therapeutic factors and pathways in musculoskeletal and metabolic health.

## MATERIALS AND METHODS

### Animals.

*Zfp423^fl/fl^* mice were kindly provided by S. Warming (Genentech) (15) and *Pax7-Cre* mice (66) were obtained from Jackson Laboratories. To conditionally delete Zfp423 within the satellite cell lineage, *Zfp423^fl/fl^* mice were interbred with *Pax7-Cre* mice to obtain *Pax7-Cre; Zfp423^fl/fl^* knockout mice (Zfp423^cko^). Homozygous *Zfp423^fl/fl^* littermate mice were used as controls. All strains were maintained on a C57BL/6N genetic background. All animal protocols were in compliance to the Harvard Medical School Institutional Animal Care and Use Committee policies.

### Plasmids.

pBICEP-3xFLAG-Zfp423 was cloned into pBICEP-3xFLAG empty vector (Sigma) as previously described (13). The 3xFLAG-GFP expression plasmid was obtained from Genecopoiea. Satb2 expression plasmid was obtained from the Harvard Medical School PlasmID Repository. Zfp423 expression plasmid (pCMV6-Kan/Neo-Zfp423-MycFLAG), empty plasmid (pCMV6-Kan/Neo), Zfp423 shRNA (sequences: ACCGCTGTCCTGGAGATGGTGATGACGAC, CAGGACACCTCTCCGCAGAAGGCTTGCCG, GATGTGATTGCTTGGCTATTGTCTGAATA, and GCGATCACTGTCAGCAGGACTTCGAGTCT) and scrambled shRNA control (TR30015) were obtained from Origene. The BMP reporter plasmid (IDWT-luc) containing BMP response elements from the ID1 promoter was kindly provided by T. Katagiri (Saitama Medical University) (48). SBE4-Luc was a gift from Bert Vogelstein (Addgene plasmid, catalog no. 16495) (67). pcDNA-FLAG-Satb2 was obtained from GenScript.

### Cardiotoxin injury model.

Mice were anesthetized via an intraperitoneal injection of Avertin at a dose of 25 mg/kg. Cardiotoxin (CTX) from *Naja mossambica* (Sigma) was dissolved in saline at a concentration of 10 μM. A 50-μl dose of CTX was injected into the tibialis anterior (TA) muscles of 7-week-old mice (satellite cells are quiescent at this time point [68]) with a 26-gauge needle. Animals were sacrificed 7 days after injection for histological analysis.

### Histological analysis.

Dissected TA muscles were immediately flash frozen in OCT embedding medium with liquid nitrogen or immediately fixed in 2% formaldehyde. Transverse sections (10 μm) of muscle were prepared with a cryostat. Formaldehyde-fixed samples were processed into paraffin blocks and sectioned at 5 μm. Morphological analysis was performed by staining sections with hematoxylin and eosin (H&E). For Pax7 immunofluorescence, staining was carried out with a Mouse-On-Mouse kit (Vector Labs). For immunofluorescence on cell cultures, cells were fixed with 3.7% formaldehyde, permeabilized with 0.1% Triton X-100 in phosphate-buffered saline (PBS), blocked with 3% bovine serum albumin, and then incubated with primary antibodies overnight at 4°C. After a washing step with PBS, the cells were incubated with Alexa Fluor-labeled secondary antibodies (Invitrogen) for 1 h at room temperature and then mounted with Prolong Antifade containing DAPI (4′,6′-diamidino-2-phenylindole; Invitrogen). The antibodies used were as follows: Pax7 (DSHB; catalog no. PAX7-s; dilution 1:10), Ki67 (Abcam; catalog no. ab15580, dilution 1:1,000), myosin MF-20 (DSHB; catalog no. MF20-s; dilution 1:20), Zfp423 (Millipore; catalog no. ABN410; dilution 1:250), MyoD (Santa Cruz; catalog no. sc-32758; dilution 1:100), myogenin (Santa Cruz; catalog no. sc-12732; 1:100), anti-rabbit antibody–Alexa Fluor 568 (Invitrogen; catalog no. A-11036; dilution 1:500), anti-rabbit antibody–Alexa Fluor 488 (Invitrogen; catalog no. A-11034; dilution 1:500), anti-mouse antibody–Alexa Fluor 568 (Invitrogen; catalog no. A-11031; dilution 1:500), and anti-mouse antibody–Alexa Fluor 488 (Invitrogen; catalog no. A-11029; dilution 1:500). Histomorphometric measurements and cell counting were performed with ImageJ software (National Institutes of Health) and the Keyence BZ-X700 software suite (Keyence Corp.). For myofiber analysis, a minimum of 100 fibers with five to six satellite cells per fiber was analyzed.

### Cell culture.

Sol8 and C2C12 myoblast cells (ATCC) were maintained in Dulbecco modified Eagle medium (DMEM; Gibco) supplemented with 20% fetal bovine serum (FBS; Gibco) and 1% penicillin-streptomycin (Gibco). Myoblast differentiation was induced 24 h after plating by treating confluent cells with DMEM supplemented with 2% horse serum and 1% penicillin-streptomycin. Medium was replaced every 48 h. Adipogenic differentiation was induced by treating cells with a cocktail of 1 mM dexamethasone, 5 mg/ml insulin, and 0.5 mM isobutyl methylxanthine (all Sigma) for 48 h, after which the medium was changed to complete DMEM plus insulin for another 48 h and thereafter maintained in complete DMEM. Lipid droplets were stained using 0.2% Oil Red O (Sigma) in 60% isopropanol after fixation with 10% neutral buffered formalin. Stable cell lines were produced by transfecting cells with X-tremeGENE HP (Roche) and selecting for stable clones in growth medium supplemented with puromycin (Life Technologies) or G418 (Life Technologies) for shRNA or cDNA plasmids, respectively. To isolate untouched primary satellite cells, dissected hind limbs were digested with 2 mg/ml collagenase II (Worthington) and 0.1% Dispase (Roche) for 1 h at 37°C. Tissue was then minced and triturated and filtered through a 70-μm-pore size filter. MACS was used to further enrich for satellite cells with a cocktail of antibodies conjugated to magnetic cell sorting beads from a Satellite cell isolation kit (Miltenyi Biotec). Subsequently, cells were cultured on collagen-coated plates in DMEM with 20% FBS, 2.5 ng/ml fibroblast growth factor 2 (FGF2), and 1% penicillin-streptomycin. Differentiation was induced with DMEM supplemented with 5% horse serum and 1% penicillin-streptomycin. For floating cultures of single isolated myofibers, EDL muscles were dissected from 6- to 9-week-old mice and digested in high-glucose DMEM containing 10 mg/ml collagenase type I (Wako) for 90 min at 37°C. Digested muscles were then rinsed in DMEM supplemented with 10% FBS and single myofibers liberated by gentle trituration. Intact, single, and well-isolated fibers were picked under a stereomicroscope and finally cultured in suspension in individual wells containing DMEM supplemented with 30% FBS, 1% chicken embryo extract, 10 ng/ml FGF2, and 1% penicillin-streptomycin in individual wells.

### Gene knockdown by small interfering RNA (siRNA).

Satb2 knockdown was performed with ON-TARGETplus SMARTpool siRNA (Dharmacon). The target sequences for the mixture of four Satb2 siRNAs (catalog no. L-058915-01-0005) were UGUCAGAGAUACUGCGUAA, AGUUCGUCUUGGUGCGGAA, UUGAAUACGACAACCGAGA, and CCAACUUAUGGAAGCGAU. A pool of nontargeting siRNA (catalog no. D-001810-10-05) was used was as control. Cells were fast forward transfected with 50 nM siRNA using HiPerFect (Qiagen) transfection reagent according to the manufacturer’s protocol.

### Quantitative real-time RT-PCR.

Total RNA was isolated from cells or tissue using High Pure RNA isolation kit (Roche). For tissue RNA extraction, tissue was first flash frozen with liquid nitrogen and then crushed into powder with a handheld homogenizer. RNA was reverse transcribed using a Transcriptor first strand cDNA kit (Roche) and real-time PCR carried out on an iCyclerIQ thermocycler (Bio-Rad) using FastStart Universal SYBR Master Mix (Roche). Relative quantification of gene expression was performed by the Δ*C_T_* method using *Gapdh* and *Tbp* as the housekeeping genes for normalization. The primer (Integrated DNA Technologies) sequences are available upon request.

### Luciferase reporter assays.

Luciferase reporter plasmids, along with Zfp423 or Satb2 expression plasmids, were cotransfected into HEK293 cells transfected using XtremeGene HP (Roche), and dual luciferase activity was assessed using a Dual-Glo assay system (Promega). Renilla luciferase (pRL-TK) (Promega) was used as an internal control for transfection efficiency. To examine BMP2 responses, cells were maintained in 0.5% FBS and treated with 100 ng/ml BMP2 (R&D Systems) or vehicle 16 h prior to assay.

### Preparation of nuclear extracts, immunoprecipitation, and mass spectrometry.

C2C12 cells stably expressing 3×FLAG-Zfp423 were washed twice with cold PBS and scraped into PBS. After centrifugation, the cell pellet was resuspended in four pellet volumes of ice-cold hypotonic buffer A (10 mM HEPES [pH 7.5], 1.5 mM MgCl_2_, and 10 mM KCl). After a 15-min incubation on ice, cells were lysed with a Dounce homogenizer, and nuclei were pelleted by centrifugation. The nuclei were then washed twice in buffer A and resuspended in half a nuclear pellet volume of 20 mM HEPES (pH 7.5), 1.5 mM MgCl_2_, 20 mM KCl, and 25% glycerol. Proteins were released by the dropwise addition of high-salt buffer (20 mM HEPES, 1.5 mM MgCl_2_, 1 M KCl, and 25% glycerol) to a final salt concentration of 300 mM KCl and gently stirred for 30 min. Nuclear debris were cleared by ultracentrifugation, and the nuclear protein supernatant dialyzes into buffer D (20 mM HEPES, 150 mM KCl, 0.05% NP-40, and 10% glycerol). All buffers were supplemented with phenylmethylsulfonyl fluoride (Sigma) and a protease/phosphatase inhibitor cocktail (Calbiochem). All steps were carried out in a 4°C cold room.

To purify Zfp423 complexes for MS, anti-FLAG M2 magnetic beads (Sigma; catalog no. M8823) were added to the nuclear extracts, followed by incubation for 3 h at 4°C. Beads were washed five times with buffer D, then washed with buffer D supplemented with 500 mM KCl, and then washed one final time with buffer D. Bound proteins were eluted by incubating the beads with 0.4 mg/ml 3×FLAG peptide dissolved in buffer D. Eluted proteins were precipitated with trichloroacetic acid and analyzed by LC-MS/MS at the Taplin Biological Mass Spectrometry Facility at Harvard Medical School. From the raw data, proteins with ≥3 peptide matches were considered confidently identified. Common background proteins such as keratins were removed. High-confidence interaction proteins were further resolved by the removal of ribosomal proteins, and selection criteria restricted to nuclear (GO:0005634) and DNA-binding (GO:0003677) transcriptional regulators.

For the validation of endogenous protein interactants, untransfected naive C2C12 cells were lysed with HKMG lysis buffer (10 mM HEPES [pH 7.9], 100 mM KCl, 5 mM MgCl_2_, 10% glycerol, 1 mM dithiothreitol, and 0.5% NP-40) containing protease and phosphatase inhibitor cocktails (Cell Signaling). Then, 1 mg of protein was incubated with anti-Zfp423 (Santa Cruz; catalog no. sc-48785), anti-Satb2 (Abcam; catalog no. ab51502) or normal IgG (Cell Signaling; catalog no. 2729) antibody conjugated to protein G-Dynabeads (Invitrogen) and incubated for 3 h at 4°C. After a washing step with lysis buffer five times, bound proteins were eluted with 2× SDS sample buffer and analyzed by Western blotting.

The antibodies used for Western blotting were as follows: anti-FLAG–horseradish peroxidase (anti-FLAG–HRP) conjugate (Cell Signaling; catalog no. 86861; dilution 1:2,000), anti-histone H3–HRP conjugate (Cell Signaling; catalog no. 12648; dilution 1:2,000), anti-Gapdh–HRP conjugate (Cell Signaling; catalog no. 8884; dilution 1:2,000), anti-Satb2 (Abcam; catalog no. ab92446; dilution 1:2,000), anti-Zfp423 (Santa Cruz; catalog no. sc-48785; dilution 1:1,000), anti-rabbit antibody (conformation specific)–HRP (Cell Signaling; catalog no. 5127; dilution 1:2,000), and anti-mouse antibody (light chain specific)–HRP (Cell Signaling; catalog no. 58802; dilution 1:2,000).

### Proliferation assays.

BrdU incorporation and quantification were carried out with a BrdU cell proliferation kit (Cell Signaling) according to the manufacturer’s instructions. Briefly, cells were seeded at a density of 5,000 cells/cm^2^ in 96-well plates, followed by incubation with 10 μM BrdU for 5 h, after which the BrdU was colorimetrically quantified with an anti-BrdU antibody-based enzyme-linked immunosorbent assay (ELISA). Proliferation analysis via DNA quantification was carried out with the CyQuant cell proliferation assay kit (Life Technologies) according to the manufacturer’s instructions.

### Statistical analysis.

Data are presented as means ± the standard deviations (SD). Comparisons were made using independent samples, unpaired Student's *t* test, or two-way analysis of variance (ANOVA) with a *post hoc* test as indicated. Statistical significance relative to indicated controls is represented by asterisks. Experiments were performed in triplicate. For *in vivo* experiments, ≥4 mice per genotype were analyzed.

## Supplementary Material

Supplemental file 1
